# Transcriptome analyses of nine endocrine tissues identifies organism-wide transcript distribution and structure in the Siberian hamster

**DOI:** 10.1038/s41598-022-16731-0

**Published:** 2022-08-08

**Authors:** Calum Stewart, Graham Hamilton, Christopher J. Marshall, Tyler J. Stevenson

**Affiliations:** 1 Institute of Biodiversity, Animal Health and Comparative Medicine, University of Glasgow, Glasgow, G61 1QH UK; 2 Glasgow Polyomics, Wolfson Wohl Cancer Research Centre, University of Glasgow, Glasgow, UK

**Keywords:** Zoology, Endocrinology, Transcriptomics

## Abstract

Temperate zone animals exhibit seasonal variation in multiple endocrine systems. In most cases, peripheral organs display robust switches in tissue involution and recrudescence in mass. Our understanding of the molecular control of tissue-specific changes in seasonal function remains limited. Central to this problem is the lack of information on the nucleic acid structure, and distribution of transcripts across tissues in seasonal model organisms. Here we report the transcriptome profile of nine endocrine tissues from Siberian hamsters. Luteinizing hormone receptor expression was localized to gonadal tissues and confirmed previous distribution analyses. Assessment of the prolactin receptor reveal relatively high abundance across tissues involved in reproduction, energy, and water homeostasis. Neither melatonin receptor-1a, nor -1b, were found to be expressed in most tissues. Instead, the closely related G-protein coupled receptor *Gpr50* was widely expressed in peripheral tissues. Epigenetic enzymes such as DNA methyltransferase 3a, was widely expressed and the predominant DNA methylation enzyme. Quantitative PCR analyses revealed some sex- and tissue-specific differences for prolactin receptor and DNA methyltransferase 3a expression. These data provide significant information on the distribution of transcripts, relative expression levels and nucleic acid sequences that will facilitate molecular studies into the seasonal programs in mammalian physiology.

## Introduction

Seasonal rhythms in mammalian endocrine function are ubiquitous in temperate and equatorial species^1^. Animals have evolved mechanisms to orchestrate the timing of physiological processes across peripheral organs to ensure life history transitions in reproduction, energy balance, and immune function^2,3^. The Siberian hamster (*Phodopus sungorus*) is a key mammalian model for studies that seek to understand the molecular basis of seasonal physiology^4,5^. In laboratory conditions, a change from summer-like long photoperiods to winter-like short photoperiods induces involution of reproductive tissues^6–9^, energy stability (i.e., adipose^6,7,9–11^, decreased body temperature^12^, change in pelage color^6^, and enhanced immunity^13–17^). Tissue involution has also been documented in a range of other peripheral tissues such as spleen, liver, brown adipose, adrenal gland, and kidney^18^.

The physiological code to drive mammalian seasonality in endocrine function is derived from the nocturnal duration of melatonin secretion from the pineal gland^19,20^. The distribution of central melatonin receptors using in vitro autoradiographic and in situ hybridization has identified binding sites in the hypophyseal pars tuberalis, and the suprachiasmatic nucleus in the hypothalamus^21–23^. Three melatonin receptor isoforms have been characterized and referred to as *Mtnr1a*, *Mtnr1b*, and *Mtnr1c*^24^. *Mtnr1a* expressing cells have been identified in the suprachiasmatic nucleus of the hypothalamus and co-expressed in vasopressin neurons, a cellular population critical for the maintenance of daily rhythms^25^. Targeted knockout of *Mtnr1a* in melatonin-sensitive mice impacted photoperiodic-induced changes in deiodinase-type2 expression, a key gene in the neuroendocrine control of reproduction across seasonal states^26^. In the Siberian hamster, there are two nonsense mutations in the coding region of the *Mtnr1b* which renders the protein non-functional and consequently ineffective for signalling nocturnal duration^27^. The third melatonin receptor is a nuclear receptor that is a melatonin sensitive form of the quinone reductase 2 and is widely distributed across endocrine tissues, however the link with seasonal rhythmicity in molecular pathways is unclear^28^. An ortholog of *Mtnr1c* is a G-protein coupled receptor 50 (*Gpr50*)^29^ that is expressed in the ependymal layer of the third ventricle and is a critical modulator of thermogenesis and torpor in mice^30^. In Siberian hamsters, *Gpr50* is also expressed in the ependymal layer and the expression level is significantly reduced in short day reproductively regressed states^31,32^. Most research has focused on the mechanisms of melatonin action in the brain, this is in part due to a lack of knowledge on the distribution of *Mtnr1a*, *Mtnr1b,* and *Gpr50* in peripheral tissues. Other hormonal signals derived from the brain and/or peripheral tissues provide an additional layer of complexity for inter-organ communication required to support seasonal changes in physiological processes^33,34^. A clear example is the annual change in gonadal steroids, such as oestradiol and progesterone in females and testosterone in males. The distribution of most hormone receptors (e.g., progesterone receptor (*Pgr*)) in peripheral tissues has yet to be characterized in highly seasonal species.

RNA-sequencing approaches provide a valuable method to identify transcript sequence, relative abundance, and distribution across tissues^35^. Our group has recently led efforts to sequence the Siberian hamster genome and delineate the hypothalamic transcriptome providing a significant gain for molecular studies in Siberian hamsters^36^. The main objective here was to sequence the transcriptome of nine peripheral tissues in Siberian hamsters. The aim was to develop an organism-wide map of the distribution and nucleic-acid sequence of all transcripts expressed in these tissues. qPCR assays were conducted to examine sex- and tissue-specific expression levels of prolactin receptor (*Prlr*), proopiomelanocortin (*Pomc*), deiodinase type-3 (*Dio3*), and DNA methyltransferase 3a (*Dnmt3a*) transcript expression. *Prlr, Pomc, Dio3, and Dnmt3a* were selected for target expression due to either the wide tissue distribution identified by RNA-sequencing analyses (i.e., *Prlr*) or established roles in the neuroendocrine regulation of seasonal physiology in Siberian hamsters (*Pomc*^36,37^, *Dio3*^9,11^ and *Dnmt3a*^9,38^). The findings reported below establish the distribution of enzyme and hormone receptors critical for thyroid, prolactin, progesterone, and sex-steroids and identified new patterns of expression.

## Results

Given the goal of this transcriptomic analysis was to provide a broad survey of tissue-specific transcripts, RNA sequencing was conducted using male and female hamsters. Non-gonadal tissues from male and female animals were pooled for sequencing, while gonadal tissues from the two sexes were sequenced separately. Illumina sequencing identified total transcript numbers of 40,729 (liver), 40,175 (brown adipose tissue), 46,383 (white adipose tissue), 47,326 (testes), 50,301 (ovary), 55,817 (uterus), 49,992 (adrenal), 45,938 (kidney), and 47,471 (spleen) in hamsters (Table S1). Of these transcripts, those with relatively more abundant counts per million (i.e., > 100) were 1536 (liver), 1423 (brown adipose tissue), 1477 (white adipose tissue), 1848 (testes), 1394 (ovary), 1413 (uterus), 1503 (adrenal), 1740 (kidney) and 1066 (spleen). Functional analyses using DAVID of gene list comprising the top 400 transcripts identified several anticipated tissue-specific gene pathways (Table 1, Table S2). In the liver, genes were closely associated with gene ontology terms identified as ‘degradation of aromatic compounds’, ‘fatty acid metabolism’ and ‘fatty acid degradation’. Both brown- and white-adipose tissue identified the ‘citrate cycle’, and ‘tissue-specific pathways’. In testes, ovary and uterine tissues, ‘oestrogen signalling pathway’ was a common and highly enriched functional pathway. Other important terms identified in the uterus were ‘vascular smooth muscle contractions’ and ‘oxytocin signalling pathways’, both implicated in the endocrine regulation of parturition. The kidneys were highly enriched in genes involved in ‘proximal tubule bicarbonate reclamation’, a key function to maintain water balance. Lastly, functional pathways found in the spleen were associated with ‘antigen processing and presentation’, ‘leukocyte transendothelial migration’ and ‘platelet activation’.

**Table 1:** Representative Gene ontology terms from nine peripheral organs in Siberian hamsters.

Endocrine system	Term	Fold enrichment
**Energy balance**		
Liver	Degradation of aromatic compounds	36.2
	Fatty acid metabolism	9.07
	Fatty acid degradation	8.88
Brown adipose	Citrate cycle (TCA)	32.4
	Fatty acid degradation	14.5
	Carbon metabolism	12.9
	Oxidative phosphorylation	11.3
White adipose	Glyoxylate and dicarboxylate metabolism	8.88
	Citrate cycle (TCA)	6.25
	Biosynthesis of amino acids	5.94
	Glutathione metabolism	5.55
**Reproduction**		
Uterus	Vascular smooth muscle contraction	5.27
	Estrogen signaling pathway	4.83
	Oxytocin signaling pathway	4.58
	GnRH signaling pathway	4.52
Ovary	Aldosterone synthesis and secretion	5.16
	Oxytocin-signaling pathway	4.88
	Estrogen signaling pathway	4.29
Testes	Glycolysis/Gluconeogenesis	7.60
	Estrogen signaling pathway	5.17
**Water balance and immune function**		
Kidney	Citrate cycle (TCA)	15.0
	Proximal tubule bicarbonate reclamation	11.3
	Mineral absorption	5.50
Adrenal	Aldosterone synthesis and secretion	4.75
	Estrogen signaling pathway	4.74
Spleen	Antigen processing and presentation	4.20
	Leukocyte transendothelial migration	3.41
	Platelet activation	2.95

### Identification of tissue-specific transcript expression profiles

**Figure 1:**
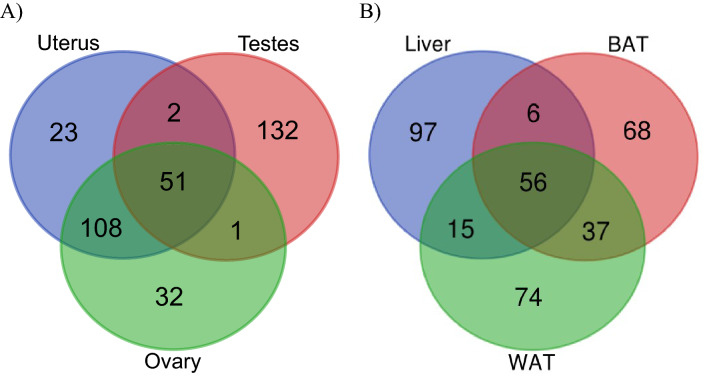
Venn diagrams identify common transcripts in tissues involved in reproduction or energy homeostasis. (**A**) Highly expressed transcripts (i.e., top 200) from testes, uterus, and ovary transcriptomes were compared. Testes has a large number of tissue-specific transcripts (i.e., 132); whereas the ovary and uterus had high overlapping transcripts (i.e., 108). (**B**) Overall, the liver, white- and brown-adipose had more tissue-specific transcripts accounting for 97, 74 and 68; respectively. There were 56 of the 353 transcripts that overlapped across all three tissues.

Venn diagrams were used to identify common, and tissue specific expression of transcripts involved in reproduction and energy balance (Fig. 1) (Table S3). We selected the top 200 transcripts from reproductive tissues (i.e., uterus, testes and ovary) and energy balance (i.e., liver, brown- and white-adipose). Using these relatively more abundant transcripts identified that 132 transcripts were specific to the testes, with 108 transcripts overlapping in the uterus and ovary (Fig. 1A). There was relatively little tissue-specific transcript expression with only 23 identified in the uterus and 32 observed in the ovary. There was considerably less overlap when comparing liver, brown- and white-adipose tissue (Fig. 1B). The liver had 97, brown adipose tissue had (68) and white-adipose tissue had (74) localized transcripts. Gene ontology analyses of overlapping transcripts revealed that only ATP binding was conserved across (1) testes, ovary and uterus, and (2) liver, brown- and white-adipose tissue (both had 3 counts each P < 0.02).

### Distribution of transcripts involved in reproduction, energy balance and epigenetic plasticity

**Figure 2:**
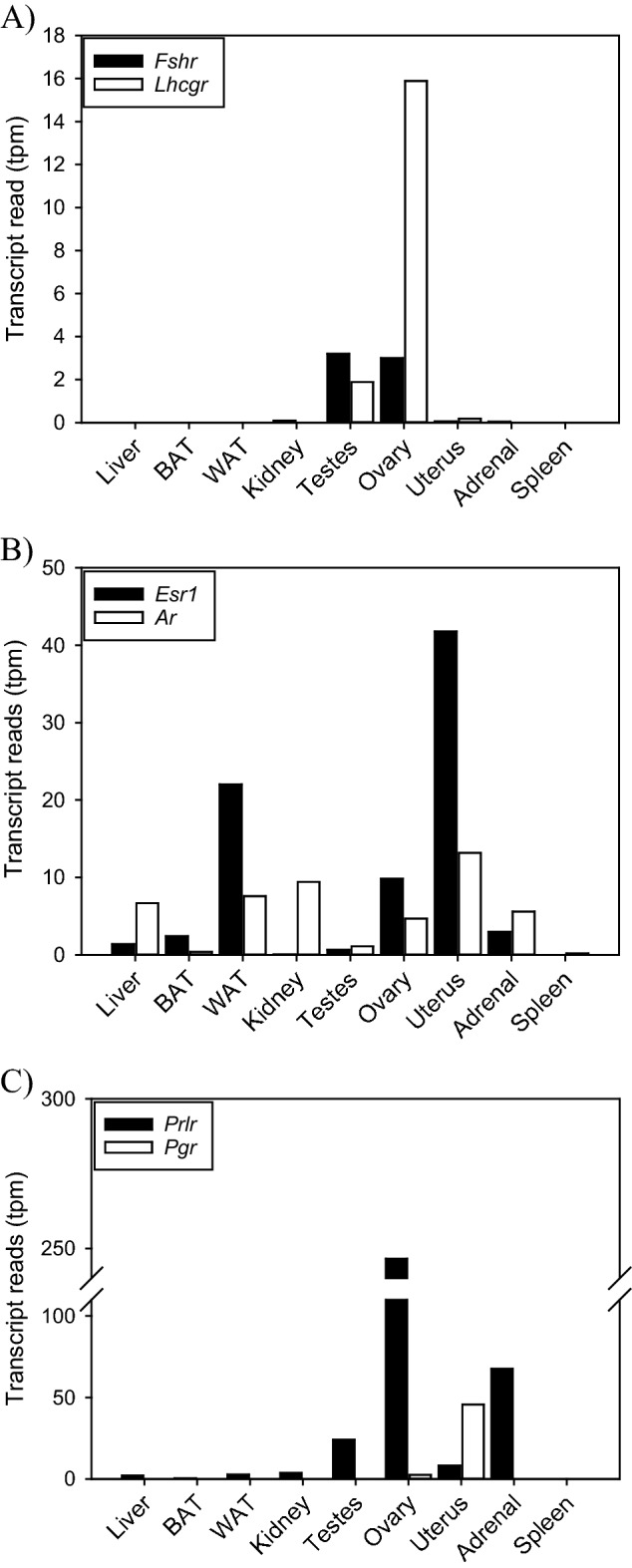
Transcript expression of receptors involved in reproduction. (**A**) Luteinizing hormone receptor (*Lhcgr*) and follicle stimulating hormone receptor (*Fshr*) transcript abundance. (**B**) Tissue expression of estrogen receptor-1 (*Esr1*) and androgen receptor (*Ar*). (**C**) Organism-wide expression of prolactin receptor (*Prlr*) and progesterone receptor (*Pgr*) in Siberian hamster tissues.

Next, we examined the number of transcript reads involved in key endocrine process across all nine tissues. Our analyses confirmed that follicle-stimulating hormone receptor (*Fshr*) and luteinizing hormone receptor (*Lhcgr*) expression was localized to gonadal tissue, with very low levels detected in the uterus (Fig. 2A). Oestrogen receptor α (*Esr1*) and androgen receptor (*Ar*) were widely distributed and observed across all tissues with the highest levels found in ovary and uterus, as well as white-adipose tissue (Fig. 2B). Unlike *Esr1 and Ar*, progesterone receptor (*Pgr*) has a restricted expression profile and was predominantly localized to the ovary and uterus (Fig. 2C). Given the role of prolactin as a read out of circannual timing by the pituitary gland, we examined the distribution of the prolactin receptor (*Prlr*) (Fig. 2C). We discovered that *Prlr* is widely expressed in reproductive tissues (i.e., testes, ovary and uterus) but also in tissues associated with energy balance and water homeostasis (i.e., kidney, adrenal). These findings raise the tantalizing hypothesis that highly seasonal prolactin secretion from the pituitary coordinates multiple physiological processes.

**Figure 3:**
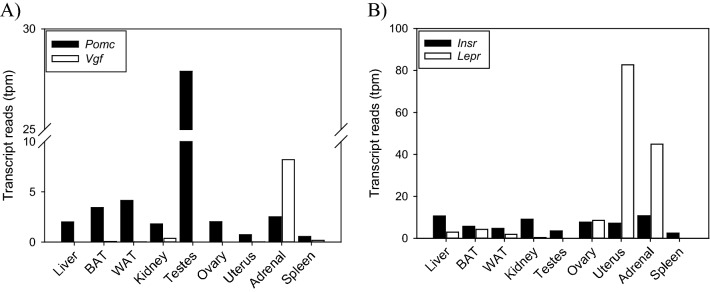
Transcript expression of neuropeptides and receptors involved in energy balance. (**A**) Proopiomelanocortin (*Pomc*) expression was found across all tissues with higher transcripts per million in the testes. VGF nerve growth factor inducible (*Vgf*) had a limited distribution and observed in the kidney and adrenal gland. (**B**) Insulin receptor (*Insr*) was across all tissues. Leptin receptor (*Lepr*) had relatively high transcripts per million in the uterus and adrenal gland and relatively lower transcripts per million in the liver, brown- and white-adipose tissue.

As proopiomelanocortin (*Pomc*) and VGF nerve growth factor inducible (*Vgf*) are precursor peptides that are subsequently cleaved into multiple peptides involved in central control of physiology^37^, we examined their expression profiles across peripheral tissues (Fig. 3A). *Pomc* has a remarkable expression profile with high transcript counts per million in the testes, but also identified across all tissues analysed. These data are exciting and indicate that molecules derived from *Pomc* likely have autocrine and/or paracrine effects within peripheral tissues. Melanocortin receptor -3 and -4 expression in the periphery was completely absent aside from a very low level of expression in the uterus. *Vgf* expression had a restricted distribution and was limited to the adrenal, kidney, and spleen. Other transcripts involved in energy balance such as insulin receptor (*Insr*) and leptin receptor (*Lepr*) displayed low expression levels across most tissues (Fig. 3B). *Lepr* expression in the uterus and adrenal gland was considerably higher than other tissues highlighting leptin signalling in these tissues as a possible avenue for seasonal physiology research.

**Figure 4:**
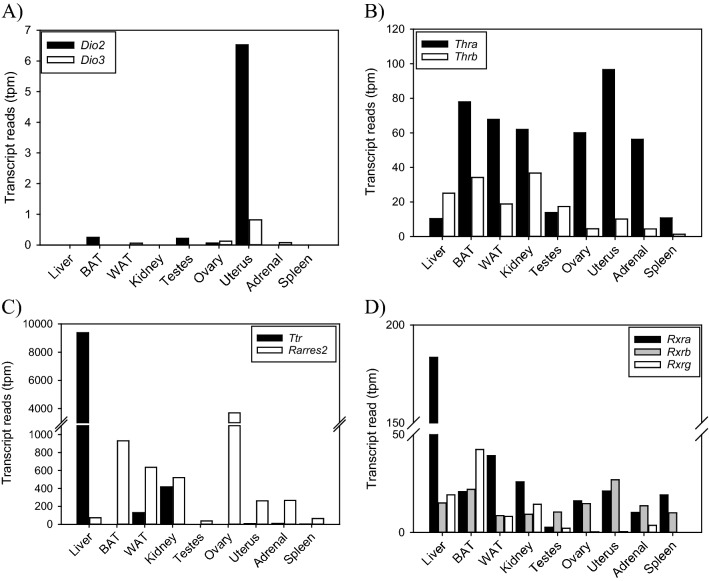
Transcript expression of genes involved in thyroid and retinoic acid signalling. (**A**) Deiodinase type-2 (*Dio2*) and type-3 (*Dio2*) expression was rarely detected in most tissues, except for the uterus. (**B**) Thyroid hormone receptor-α (*Thrα*) and -β (*Thrβ*) were identified across most tissues with *Thrα* as the predominant isoform. (**C**) Transthyretin (*Ttr*) had a limited distribution and localized to the liver, white-adipose and kidney. (*Rarres2*) was more widely distributed with high expression in adipose tissues, kidney, ovary, uterus, adrenal gland and spleen. (**D**) Retinoic acid receptor-*α* (*Rxrα*), -*β* (*Rxrβ*), and -γ (*Rxrγ*) were expressed across all tissues. *Rxrα* is the predominant isoform and had higher transcripts per million in the liver.

Assessment of thyroid hormone enzymes, *Dio2* and *Dio3* shows presence of expression to uterine tissue, with very low levels of *Dio2* in testes, ovary, and brown-adipose tissue (Fig. 4A). Conversely, thyroid hormone receptors-α (*Thrα*) and -β (*Thrβ*) are widely distributed (Fig. 4B). *Thrα* was most abundantly expressed in all tissues with high levels in adipose tissue, kidney, adrenals, ovary and uterus. *Thrβ* expression had lower expression identified primarily in liver, adipose kidney, and testes. The thyroid and retinoic acid transporter, transthyretin (*Ttr*) was localized to the liver, white adipose tissue, and kidney, whereas the retinoic acid receptor responder 2 (*Rarres2*) was identified across all tissues with relatively abundant transcripts per million in adipose, kidney, ovary, uterus, and adrenals (Fig. 4C). The three retinoic acid receptors were also identified in all tissues with more abundant transcripts per million, except for the gamma isoform (*Rxrγ*) which had lower counts per million in the ovary, uterus, and spleen (Fig. 4D). Lastly, transcriptomes were searched for the two melatonin receptors (*Mtnr1a*, *Mtnr1b*) with surprisingly little identification across tissues. Only the uterus expressed the *Mtnr1a* isoform with only 2 transcripts confirmed. Conversely, the melatonin-sensitive G Protein-Coupled Receptor-50 (*Gpr50*) expression was found in the liver, kidney, white-adipose tissue and adrenal.

### Distribution and expression level of transcripts involved in epigenetic modifications

**Figure 5:**
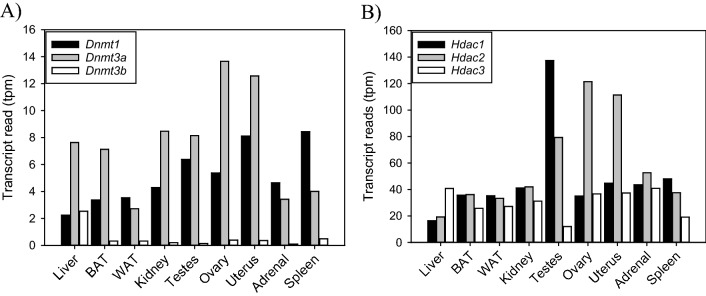
Epigenetic enzyme expression in Siberian hamster tissues. (**A**) DNA methyltransferase 1, (*Dnmt1*), 3a (*Dnmt3a*) and -3b (*Dnmt3b*) were identified in all tissues. The maintenance *Dnmt1*, and de novo DNA methylation enzymes *Dnmt3a* had relatively high transcripts per million. (**B**) Three histone deacetylase (*Hdac*) enzymes were selected for analyses and were identified in all tissues. Most tissues were found to expression each transcript with relatively higher counts per million for *Hdac1* in testes and *Hdac2* in ovary and uterine tissues.

DNA methyltransferase (*Dnmt*) and histone deacetylase (*Hdac*) enzyme expression was characterized across the peripheral tissues (Fig. 5). Both maintenance *Dnmt1* and de novo* Dnmt3a/3b* enzymes are expressed in all tissues, however *Dnmt1* and *Dnmt3a* are the predominant enzymes (Fig. 5A). Similarly, *Hdac1-3* enzymes are ubiquitously expressed in hamster tissues (Fig. 5B). *Hdac1* showed highest counts per million in the testes, whereas *Hdac2* was more abundant in the ovary and uterus, suggesting these enzymes have gonad-specific reproductive functions.

### Sex comparison of *Prlr, Pomc, Dio3, *and* Dnmt3a* in peripheral non-gonadal tissues

**Figure 6:**
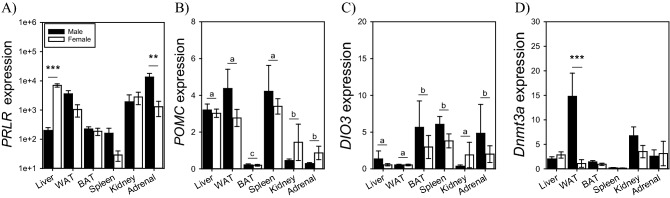
Sex comparison of *Prlr*, *Pomc*, *Dio3*, *Dnmt3a*, in non-gonadal peripheral tissues. qPCR assays were conducted to examine *Prlr*, *Pomc*, *Dio3*, and *Dnmt3a* expression in male and female tissues. (**A**) *Prlr* expression was significantly higher in female livers and male adrenal glands. (**B**) *Pomc* expression was highest in liver, white-adipose tissue (WAT), and spleens, compared to kidney and adrenal glands, and lowest levels in brown adipose tissue (BAT). Asterisks denote significant sex differences (**P < 0.01; ***P < 0.001). Letters denote significant differences between tissues (P < 0.001).

Next, to examine sex differences in transcript expression in non-gonadal tissues, we conducted qPCR assays of four genes of interest that include *Prlr, Pomc, Dio3 and Dnmt3a*. There was a significant interaction for *Prlr* (F_5,52_ = 7.59; P < 0.001), main effect of tissue (F_5,52_ = 11.43; P < 0.001), but no main effect of sex (F_1,52_ = 0.55; P = 0.46) (Fig. 6A). Fisher’s LSD identified that females had higher liver *Prlr* compared to male hamsters. Conversely, males had higher adrenal *Prlr* compared to female hamsters. *Pomc* was found to be significantly different across tissues (F_5,60_ = 442; P < 0.001), but there was no significant main effect of sex (F_1,60_ = 0.79; P = 0.37), or interaction (F_5,60_ = 1.20; P = 0.32) (Fig. 6B). The highest levels of *Pomc* expression were identified in the liver, white adipose tissue, and spleen, with moderate levels in the kidney and adrenal gland, and the lowest levels in BAT (P < 0.001 for all level comparisons). There was no significant main effect of sex on *Dio3* expression (F_1,55_ = 0.03; P = 0.84), and no significant interaction (F_5,55_ = 0.35; P = 0.87) (Fig. 6C). There was a significant main effect of tissue (F_5,55_ = 6.19; P < 001). Post-hoc analyses established that the adrenal gland, spleen, and brown adipose tissue had higher *Dio3* expression compared to the liver, white adipose tissue, and kidney. There was no significant difference between brown adipose tissue, spleen, and the adrenal gland. There was no significant difference between liver, white adipose tissue, and the kidney. There were significant main effects of sex (F_1,60_ = 6.79; P < 0.01), tissue (F_5,60_ = 12.19; P < 0.001) and an interaction (F_5,60_ = 3.33; P < 0.01) for *Dnmt3a* expression (Fig. 6D). Post-hoc analyses identified that white adipose tissue *Dnmt3a* expression was higher in males compared to females. All other tissues had similar *Dnmt3a* expression levels in both sexes (P > 0.43).

## Discussion

In this study, we report the transcriptome profiles for nine peripheral tissues from a highly seasonal rodent: the Siberian hamster. Using Illumina sequencing, our findings establish the distribution of transcripts in multiple tissues involved in reproduction, energy and water homeostasis, stress, and immune function. These data confirm previous research on receptor distributions such as high luteinizing hormone receptor and estrogen receptor in testes^39^ and ovary tissue^40^. Our approach also provided the ability to uncover that melatonin receptors *Mtnr1a* and *Mtnr1b* are not widely distributed, instead, the melatonin-insensitive receptor, *Gpr50* expression was identified in all tissues. We also report sex differences in two transcripts of interest (i.e., *Prlr*, *Dnmt3a*) that were tissue specific based on qPCR analyses. Females had significantly higher *Prlr* expression in livers, whereas males had more adrenal *Prlr* and white adipose tissue *Dnmt3a* expression.

Prolactin secretion from the pars distalis in the anterior pituitary gland provides an endogenous circannual read-out from the brain^41,42^. In mammals, prolactin has been found to be involved in short-term homeostatic regulation of water and electrolyte balance, growth and development, metabolism, reproduction and immunity^43^. Distribution of prolactin receptor expression has focused on testicular, ovarian and uterine tissue in hamsters^44,45^. The findings here show relatively high transcripts per million in ovarian tissue, and abundant expression in the testes and uterus. Prolactin receptor expression was also discovered in the liver, white-adipose tissue, kidney, and adrenal glands. This wide pattern of expression indicates that seasonal cycles of prolactin secretion may impact multiple physiological processes and is consistent with the pleiotropic nature of the hormone. Increased *Prlr* expression determined by qPCR analyses in female liver may provide a sex-specific role that links reproduction and glucose homeostasis associated with pregnancy and lactation. Functional manipulations of circulating prolactin using subcutaneous injections to white-pelage Siberian hamster housed in short photoperiods induced coat color change into the summer agouti phenotype^46^. The range of tissues expressing prolactin receptor indicates that lactotroph signalling may impact a wider range of seasonal physiological programs than previously anticipated.

Thyroid hormones action in the brain is critical for timing seasonal transition in physiology^47,48^. The localized increase in triiodothyronine in the ependymal layer of the third ventricle is necessary for the vernal increase in photoperiod induction of reproduction^49^. Deiodinase enzymes Type-2 and -3 are photoperiodically regulated in the ependymal layer of the third ventricle to govern hypothalamic concentrations of triiodothyronine. Short days induce a significant increase in *Dio3* in Siberian hamsters^9,50^. In this study, we found that both *Dio2* and *Dio3* had relatively low transcripts per million observed across endocrine tissues. Only the uterus showed relatively high transcript counts of *Dio3* expression possibly indicating differential mechanisms between sexes for the role of local triiodothyronine on gonadal function. Conversely, there was abundant transcript counts per million of the thyroid hormone receptor-alpha across tissues, with lower expression observed for the beta-subunit. These data indicate that circulating thyroid hormones impact endocrine processes and not necessarily the local conversion of thyroxine into triiodothyronine.

Retinoic acid signalling in the ependymal layer of the third ventricle is another mechanism involved in the neuroendocrine control of seasonal physiology^51,52^. Several genes involved in retinoic acid pathways are expressed in the mediobasal hypothalamus and exhibit photoperiod regulation including transthyretin, retinaldehyde dehydrogenase 1, and retinoic-acid receptors^53–55^. We searched the transcriptomes and determined that transthyretin and retinaldehyde dehydrogenase-2 show tissue-dependent expression. Transthyretin had relatively higher transcript per million counts in liver, with lower abundance in white-adipose tissue and kidney. Whereas retinaldehyde dehydrogenase-2 displayed high transcript counts in most tissues examine except the liver, and testes. Our findings also indicate that Retinoic acid receptor (*Rxr*)-α and -β are expressed in most tissues, and *Rxrα* has high levels in liver and white-adipose tissue and kidneys. The abundance of retinoic acid receptor expression across tissues suggest that metabolites of vitamin A may provide a signal to peripheral tissues to time seasonal life history transitions in physiology.

Seasonal timing of reproductive physiology has been linked to cyclical changes in epigenetic modifications such as DNA methylation and histone acetylation^38^. In ovarian, testes and uterine tissue, *Dnmt3a* and *Hdac2* are elevated during periods of seasonal infertility^56,57^. Ovarian steroids oestradiol and progesterone can, within 12 h, significantly inhibit uterine *Dnmt3a* expression. Here, *Dnmt3a* and *Dnmt1* expression are expressed in all tissues examined with relatively higher counts per million compared to *Dnmt3b*. Furthermore, *Hdac* enzymes exhibit high expression across peripheral tissues; *Hdac1* had high levels in the testes, whereas *Hdac2* was high in the ovary and uterus*.* These patterns indicate that epigenetic modifications are tissue-specific and likely have gonad-specific effects for reproductive physiology. Evidence to support this conjecture can be derived from targeted analyses of *Dnmt3a* across non-gonadal tissues. Our qPCR assays identified that white adipose tissue *Dnmt3a* expression is significantly higher in male compared to female hamsters. These data indicate that epigenetic modifications associated with energy homeostasis, may play a larger role in males. However, the functional significance, if any, for elevated *Dnmt3a* in white adipose tissue remains uncharacterised.

This study sought to uncover the transcriptome profiles of nine tissues in Siberian hamsters and examine sex-specific expression levels of a few target transcripts. Importantly, using pooled RNA samples from male and female Siberian hamsters, the findings chart the entire transcript expression patterns for the liver, white- and brown-adipose tissue, kidney, testes, ovary, uterus, adrenal gland, and spleen. The findings revealed unexpected distributions of *Gpr50* and restricted levels of transcripts for *Mtnr1a* and *Mtnr1b*. We also report, to our knowledge, the first identification of sex-specific expression patterns of *Dnmt3a* in white adipose tissues. However, pooled samples for RNA-sequencing do have limitations. The lack of biological replicates of peripheral tissue and combined male and female RNA prevents the ability to conduct statistical analyses or uncover sexually dimorphic expression patterns. The main aim of the current experimental design was to delineate all transcript sequences in nine peripheral tissues and ensure male and female samples were represented. The transcript data will be useful for future functional analyses of seasonality as well as improving the Siberian hamster genome annotation. Moreover, determining the nucleic acid sequence of thousands of genes will facilitate molecular assays required to facilitate our mechanistic understanding of programmed changes in seasonal physiology.

## Materials and methods

### Animals

Six adult Siberian hamsters were used to collect peripheral tissues to obtain transcriptome profiles. Animals were derived from a colony maintained in the Veterinary Research Facility at the University of Glasgow. The room light schedule was 16 h light, 8 h dark (15L:9D) and 51 lx. The room temperature was held at 21 °C and 50% humidity. All procedures were approved by the University of Glasgow Animal Welfare and Ethics Review Board and authorized under the United Kingdom Home Licence PP5701950. The procedures were performed in compliance with the revised Animals (Scientific Procedures) Act 1986 and were conducted in accordance with the ARRIVE guidelines (https://arriveguidelines.org/).

### Tissue dissection and RNA extraction

All hamsters were individually housed and kept in the long day (15L:9D) light schedule from birth to tissue collection. Male (n = 3) and freely cycling female (n = 3) hamsters were killed 5 h after lights on using approved Schedule 1 methods of cervical dislocation and followed by severing the jugular vein. Mean and standard error of the body mass for males (45.6 g ± 1.2) and females (32.6 g ± 0.8) was typical of long photoperiod hamsters. Testes (0.8 g ± 0.1) and uterine (0.g ± 0.02) mass provided further evidence that animals were reproductively active. Liver, spleen, kidney, epididymal white adipose tissue, testes, ovary, uterus, adrenal, muscle brown adipose tissue and pancreas were rapidly dissected, and samples were pooled into 1.5 ml microfuge tubes providing a mixture of six samples from males and females. Tubes were immediately frozen by immersion in powdered dry ice and then samples were stored at − 70 °C until RNA extraction. The pooled samples ensured that the transcriptome was not biased toward any sex. Testes, ovary, and uterine tissue was pooled from 3 biological replicates. Samples were divided into two groups for RNA extraction using either Trizol (Invitrogen, cat# 15596026) or RNeasy Plus Mini kit (QIAGEN, cat# 74136). RNA quantity and quality was determined using Nanodrop and Q-bit (Agilent Bioanalyzer) analyses. RNA extracted using RNeasy Plus Mini were consistently observed to produce 260/280 values that range from 2.0 to 2.1, and optimal RIN values at 10. Muscle and pancreatic tissue did not yield sufficient RNA to permit transcriptome sequencing. Samples were kept at − 70 °C until RNA-sequencing was conducted.

### Sample preparation for Illumina RNA-sequencing and analysis

RNA-seq was performed by Glasgow Polyomics on ribosomal-depleted RNA using an Illumina NextSeq 500 platform. Paired-end sequencing was performed with a read depth of 30 million per tissue sample. RNA-seq reads were processed and trimmed to ensure low quality bases and adapter sequences were removed using FastP version 0.20.0 and mapped to the Ensembl annotation of mouse GRCm39 transcriptome using Kallisto version 0.45.1. The mouse genome is the most suitable species to obtain accurate mapping and annotated transcripts. Unfortunately, the current Siberian hamster genome lacks to the sequences depth and includes several gaps that prohibits transcript mapping. Moreover, the genome is highly fragmented, with a contig count of 1,462,128 and that the annotation of the genome is at a very preliminary stage. Our mapping of transcriptomes to the mouse genome readily achieves approximately 95% read mapping, whereas the Siberian hamster genome only identifies roughly 60–70% of transcripts. The transcripts were annotated with Ensembl gene IDs and names with TXImport version 1.18.0 and quantified using tpm (transcripts per million). The data set was deposited in the European Nucleotide Archive accession number (PRJEB48528) (http://www.ebi.ac.uk/ena/browser/view/PRJEB48528). The biotype of each transcript was annotated according to the Ensembl database. Gene ontology to identify functional pathways was performed using DAVID Bioinformatics Resources 6.8^58,59^.

### Sex comparison in transcript expression in non-gonadal peripheral tissues

To examine sex differences in transcript expression, adult male (n = 6) and female (n = 6) hamsters were randomly selected from animals housed in the long day (16L:8D) colony room. Animals were euthanized 5 h after lights on by cervical dislocation followed by exsanguination. Liver, brown and white adipose, spleen, kidney and adrenal glands were dissected as above, rapidly frozen on dry ice and stored at -80ºC. RNA was extracted from approximately 50 mg of peripheral tissues using Trizol (ThermoFisher Scientific). RNA concentration and 260/280 values were determined by spectrophotometer to confirm nucleic acid specificity and integrity (Nanodrop; ThermoFisher Scientific). Reaction mixture contained 4 µl 200 ng/µl total RNA (800 ng total), 2 µl 5 × first strand buffer (Thermofisher Scientific), 1 µl DTT (10 mM), 0.2 µl 20 mM Random Primers (Promega), 0.2 µl 20 mM dNTP mix (Thermofisher Scientific), 0.26 µl RNasin^®^ Ribonuclease Inhibitor (Promega), 0.26 µl Superscript III reverse transcriptase (Thermofisher Scientific), 2.08 µl RNAse free water. Reaction mixture was incubated at 50 °C for 1 h. Once incubation was complete mixture was diluted with 90 µl LOTE buffer [3 mM Tris–HCl (Thermofisher Scientific), 0.2 mM EDTA (Sigma)] and cDNA was stored at − 20 °C until quantitative polymerase chain reaction (qPCR). All cDNA tissue samples were run in duplicate. qPCRs were conducted using an Agilent Stratagene mx3000p system using the following steps: (1) an initial denature at 95 °C for 30 s and then 39 cycles of (2) 95 °C for 10 s, (3) annealing dependent on target mRNA (see Table 2) for 30 s, and (4) an extension at 72 °C for 30 s. A melting curve analysis was added to determine the quality and specificity of each replicate reaction. Quantification of mRNA expression levels was accomplished with Agilent Brilliant II SYBR green. We used PCR Miner^60^ to calculate the reaction efficiencies and cycle thresholds. According to the MIQE guidelines, samples that had efficiency values below 0.8 or above 1.2 were excluded from analyses^61^. The expression of each target gene of interest was measured in relation the average cycling time of two 18S ribosomal RNA (18 s) replicates. 18S levels did not vary between sexes (P > 0.05). To compare transcript expression across tissues, the average difference of target RNA, and reference RNA for all samples was used for the second Δ. The fold-change in expression was then calculated using 2-(ΔΔCt).

**Table 2:** Primer sequences and qPCR parameters.

Gene	Sequence (5′ to 3′)	Anneal C (°C)	Melt C (°C)
*18S*	GCTCCTCTCCTACTTGGATAACTGTG	62	80
	CGGGTTGGTTTTGATCTGATAAATGCA		
*Dio3*	CATGCTCCGCTCCCTGCTGCTTCA	58	80
	CAGGGTGCACAGACGGTTGTC		
*Dnmt3a*	CTCTGCAGGAGAGGGCAAAGAACAG	60	88
	TAGCATTCTTGTCCCCAGCATCCCC		
*Pomc*	TGGAGAGCAGACAGTGTCAGGAC	60	86
	TCTCGGTCAACGTCTGGTCGTC		
*Prlr*	GGGAGCCTCTGATACATTGC	60	81
	CAGGAGAGCGACATTTGTG		

### Statistical analyses

Sex differences in *Prlr*, *Pomc, Dio3*, and *Dnmt3a* fold-change expression was compared using 2-way ANOVA conducted using SigmaPlot 14.0. All qPCR samples were log-transformed to achieve normally distributed data. Post-hoc analyses were performed using Fisher’s LSD. Significance was determined at P < 0.05.

## Supplementary Information

Supplementary Table 1.

Supplementary Table 2.

Supplementary Table 3.

Supplementary Table 4.

## Data Availability

The sequencing datasets generated and/or analysed during the current study are available in the European Nucleotide Archive accession number (PRJEB48528) repository. The transcript, GO terms and qPCR data generated or analysed during this study are included in the supplementary information files in this published article. All datasets used and/or analysed during the current study available from the corresponding author on reasonable request.
